# Evaluation of new motorized articulating laparoscopic instruments by laparoscopic novices using a standardized laparoscopic skills curriculum

**DOI:** 10.1007/s00464-020-08086-2

**Published:** 2020-10-20

**Authors:** Daniel Uysal, Claudia Gasch, Rouven Behnisch, Felix Nickel, Beat Peter Müller-Stich, Markus Hohenfellner, Dogu Teber

**Affiliations:** 1 Medical School, University of Frankfurt, Frankfurt, Germany; 2 Department of Urology, Heidelberg University Hospital, Im Neuenheimer Feld 420, 69120 Heidelberg, Germany; 3 Institute of Medical Biometry and Informatics (IMBI), University of Heidelberg, Heidelberg, Germany; 4 Department of General, Visceral, and Transplantation Surgery, Heidelberg University Hospital, Heidelberg, Germany; 5 Department of Urology, Staedtisches Klinikum Karlsruhe, Karlsruhe, Germany

**Keywords:** Motorized articulating laparoscopic instruments, Handheld robotic device, Laparoscopy, E-BLUS, Urology, Training

## Abstract

**Background:**

Motorized articulating laparoscopic instruments (ALI) offer more degrees of freedom than conventional laparoscopic instruments (CLI). However, a difficult learning curve and complex instrument handling are still a problem of ALI. We compared the performance of new prototypes of motorized ALI with CLI in a series of standardized laparoscopic tasks performed by laparoscopic novices. Further, usability of the new ALI was assessed.

**Methods:**

A randomized cross-over study with 50 laparoscopic novices who either started with CLI and then changed to ALI (CA) or vice versa (AC) was conducted. All participants performed the European training in basic laparoscopic urological skills (E-BLUS) with each instrument in given order. Time and errors were measured for each exercise. Instrument usability was assessed.

**Results:**

Overall, using CLI was significantly faster (CLI 4:27 min vs. ALI 4:50 min; *p*-value 0.005) and associated with fewer exercise failures in needle guidance (CLI 0 vs. ALI 12; *p*-value 0.0005) than ALI. Median amount of errors was similar for both instruments. Instrument sequence did not matter, as CA and AC showed comparable completion times. Regarding the learning effect, participants were significantly faster in the second attempt of exercises than in the first. In the needle guidance task, participants using CLI last demonstrated a significant speed improvement, whereas ALI were significantly slower in the second run. Regarding usability, CLI were preferred over ALI due to lighter weight and easier handling. Nevertheless, participants valued ALI’s additional degrees of freedom.

**Conclusion:**

Using new motorized ALI in the E-BLUS examination by laparoscopic novices led to a worse performance compared to CLI. An explanation could be that participants felt overwhelmed by ALI and that ALI have an own distinct learning curve. As participants valued ALI’s additional degrees of freedom, however, a future application of ALI could be for training purposes, ideally in combination with CLI.

**Electronic supplementary material:**

The online version of this article (10.1007/s00464-020-08086-2) contains supplementary material, which is available to authorized users.

Within the last 40 years laparoscopic surgery has had a profound influence on the development of operative medicine. Its benefits for the patient, such as shorter hospital stay, decreased blood loss and improved cosmetic appearance have been well established [1]. The surgeon, on the other hand, is faced with certain difficulties like reduced perception of depth, inverse translation of movements (fulcrum effect), limited degrees of freedom and increased physical demand during surgery [2]. Therefore, surgery with conventional laparoscopic instruments (CLI) shows a more difficult learning process compared to open surgery [3, 4]. Due to these obstacles, surgical robots, like the da Vinci® surgical system (Intuitive Surgical, Sunnyvale, California), have been developed. Robotic surgery offers a magnified 3D view and allows for a more natural instrument movement, using seven degrees of freedom as opposed to the four degrees of freedom offered by CLI. Another advantage of robotic systems is less physical strain during surgery [5, 6]. Besides its loss of haptic feedback, potential disadvantages of robots are high acquisition and maintenance costs, allowing only for a few specialized centers to implement robotic systems into their clinical practice [5, 6].

To address the discrepancy of technical limitations of CLI and high costs of robotic systems, various articulating laparoscopic instruments (ALI)—mechanical or motorized—have been developed [7, 8]. The hope is that they may offer enhanced degrees of freedom while being more cost-effective, thus making them affordable to a larger number of institutions. Until today only few studies tested motorized ALI. Most of them either used robotic-driven needle holders or the Kymerax™ system (Terumo, Tokyo, Japan) and were conducted in box trainers [7, 9–11]. In 2017, Sieber et al. investigated the performance of Kymerax™ instruments and found that despite increased precision participants performed tasks in a box trainer significantly more slowly and needed more training time compared to CLI [10]. Also, Tuncel’s study suggests that laparoscopic suturing with first-generation articulating needle holder might be more difficult to learn for laparoscopic novices [12].

Since a difficult learning curve and the complex instrument handling still seem to be a problem of ALI, we wanted to test new prototypes of motorized ALI of Karl Storz (KARL STORZ SE & Co., KG, Tuttlingen, Germany). As additional features they allow a motorized flection and 360° rotation of the tip, providing more degrees of freedom than CLI (Fig. 1). For motorization they must be connected to a designated motor unit, which allows selection of different speed modes and the instrument used. In contrast to other ALI the above-mentioned features can now be performed with full seven degrees of freedom. For more intuitive usage the new ALI pistol handle combines the trigger and buttons for tip rotation and deflection on the front of the handle, instead of opposite sides of the instrument like the Kymerax™ instruments [7, 10].

**Fig. 1:**
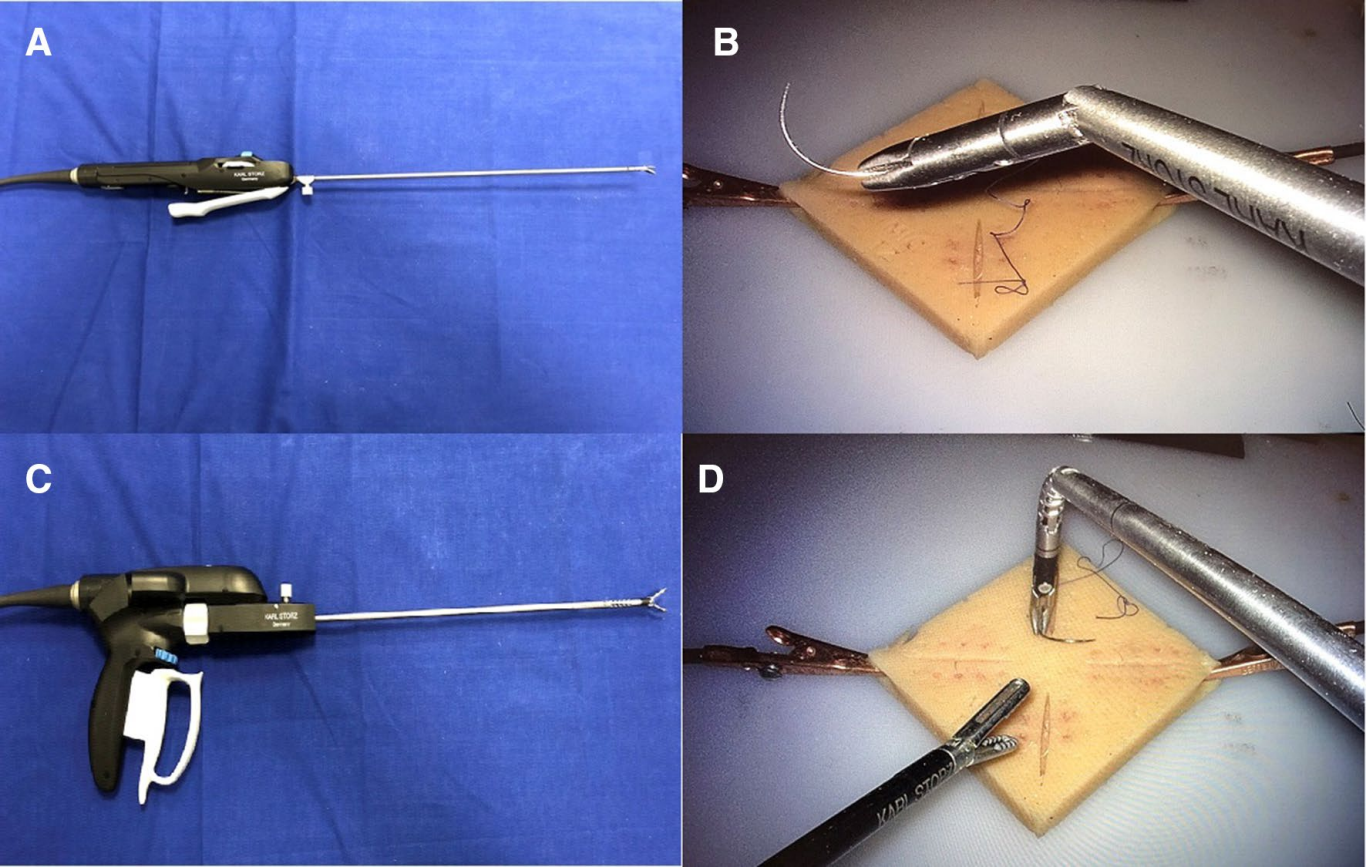
**A** Functional sample of motorized articulating needle holder: the blue and grey wheels allow rotation and flection of the instrument tip. **B** Articulating needle holder’s tip flexed and rotated. **C** Functional sample of motorized grasping forceps: the blue and grey wheels allow rotation and flection of the instrument tip. **D** Articulating grasping forceps’ tip flexed and rotated (Color figure online)

The aim of this study was to compare the performance of the new motorized ALI with CLI in a series of standardized laparoscopic tasks. The authors wanted to know whether the improvements in ergonomics and intuitiveness of the new ALI translated to equal or better results than CLI. Another aim was to assess the usability of the new ALI.

## Material and methods

### Study design

50 medical students and residents with no prior experience in laparoscopic surgery were chosen to participate in the trial (Table 1). All participants were informed about the type and extent of this study as well as the usage of gathered data and written informed consent was obtained. The study was approved by the local Ethics Committee at Heidelberg University (Code S-334/2011). The participants were randomly assigned to one of two groups in a ratio of 1:1. Group CA started the first run of the examination with CLI and then crossed over to the motorized ALI in the second run. Group AC used the instruments in reverse order.

**Table 1:** Group characteristics

	CA *n* = 25	AC *n* = 25	*p*-value
Gender			0.54
Male	16 (64%)	18 (72%)	
Female	9 (36%)	7 (28%)	
Handedness			1.00
Right	22 (78%)	22 (78%)	
Left	3 (12%)	3 (12%)	
Age			0.40
< 20	7 (28%)	3 (12%)	
20–25	16 (64%)	18 (72%)	
26–30	2 (8%)	3 (12%)	
> 30		1 (4%)	
Medical education			0.15
Preclinical	14 (56%)	7 (28%)	
Clinical	9 (36%)	13 (52%)	
Internship	2 (8%)	3 (12%)	
Resident		2 (8%)	
Hobbies			0.51
Musical instrument	9 (36%)	13 (52%)	
Video gaming	9 (36%)	6 (24%)	
Knitting/pottery	1 (4%)	2 (8%)	
Suturing skills course	1 (4%)	2 (8%)	

Differences between groups were assessed using the Chi-Square test. A *p*-value < 0.05 was regarded as statistically significant

*Group CA* Conventional laparoscopic instruments in the first, articulating laparoscopic instruments in the second run, *Group AC* Articulating laparoscopic instruments in the first, conventional laparoscopic instruments in the second run, *n* number of trainees

**Fig. 2:**
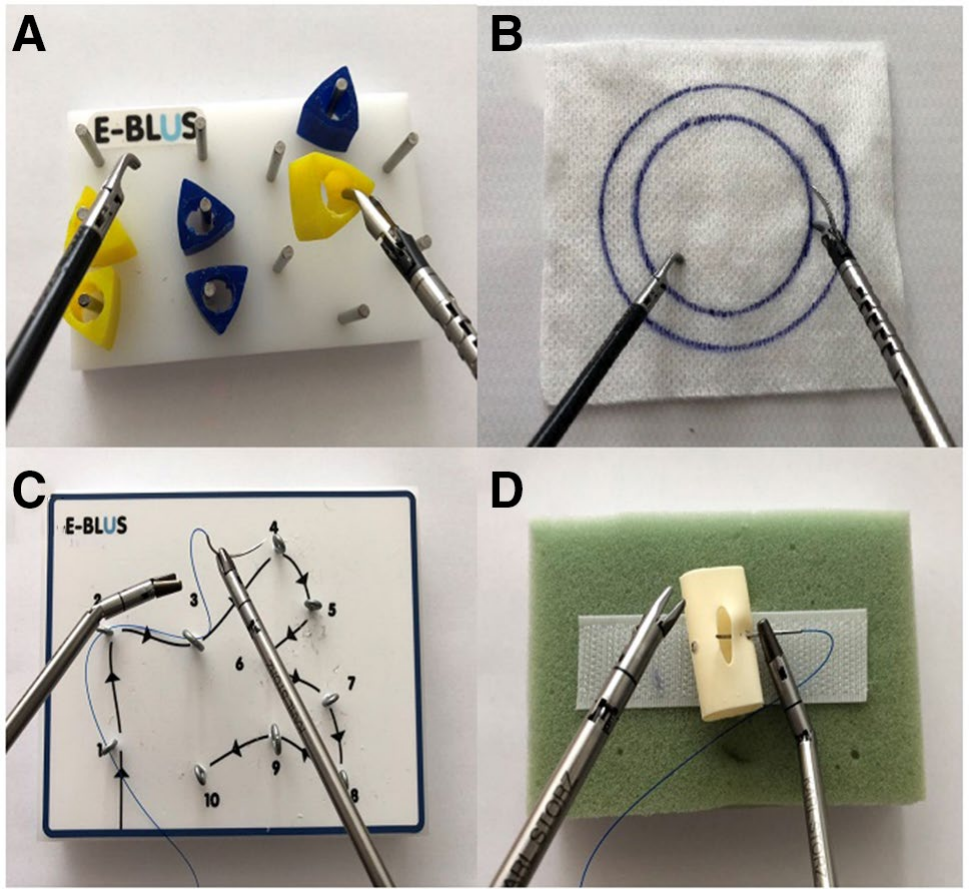
The performed E-BLUS examination tasks. **A** Peg transfer. **B** Cutting a circle. **C** Needle guidance. **D** Laparoscopic suturing

Performance with the different instruments was compared with a validated laparoscopic skills curriculum used by the European Association of Urology to certify Urology laparoscopists, the European training in basic laparoscopic urological skills (E-BLUS). The E-BLUS examination was developed to specifically evaluate the competence of basic laparoscopic skills in Urology residents and consists of four exercises: peg transfer (PT), cutting a circle (CC), needle guidance (NG), and laparoscopic suturing (LS) (Fig. 2) [13].

**Fig. 3:**
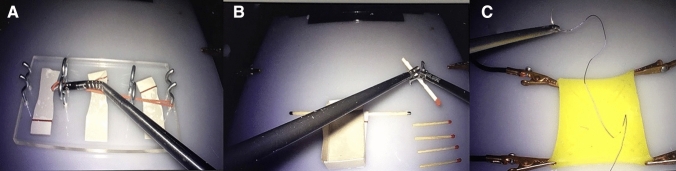
Warm-up exercises. **A** Rubber band stretch. Articulating grasping forceps’ flection allows direct advancement through the ring, which would not be possible with CLI. **B** Match transfer. Participants used ALI’s flection to pick up matches like a crane (not depicted here). **C** Laparoscopic suturing. Stitches had already been placed onto the rubber pad

Our assessment started with three warm-up tasks to familiarize participants with the different instruments (Fig. 3). Afterwards, the first E-BLUS examination was started with participants watching an official exercise video tutorial prior to each exercise (https://uroweb.org/education/online-education/surgical-education/laparoscopy/). A detailed explanation of the exercises according to Brinkman et al. can be found in the supplement [13]. After a cool-down period of 10 min when finishing the first run the process was repeated using the other instruments. A questionnaire regarding instrument usability was administered after each run (ALI or CLI). To account for the laparoscopic inexperience of participants, time tolerance of the exercises was adjusted. Time to completion, the number of mistakes and exercise failure were recorded for each exercise.

### Instrument setup

**Fig. 4:**
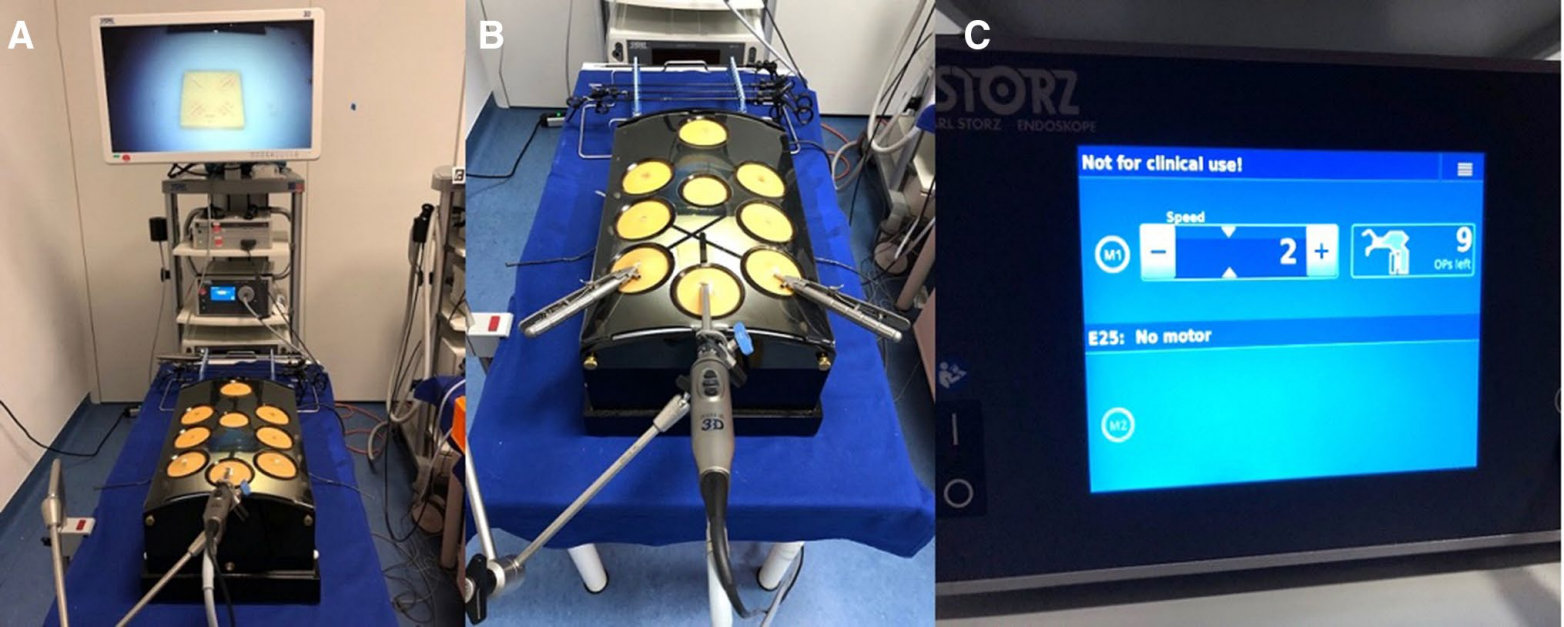
Instrument setup. **A** Laparoscopic workstation. **B** Box trainer mounted with camera and two needle holders in the trocar positions used for every exercise. **C** Motor unit of the functional samples of articulating instruments allowing to select different instruments (right side of screen) and speed (left side of screen)

To adequately simulate the conditions of operating in a pneumoperitoneum, all tasks took place in a box trainer model in 2D vision. Exercise boards and instruments were placed in the same position to allow for consistent distances (Fig. 4). Since only one motorized ALI could be attached to the motor unit at a time, participants were asked to use their dominant hand for the motorized ALI while they were given a CLI for their non-dominant hand.

Laparoscopic vision was provided using an IMAGE1 S CONNECT and IMAGE1 S D3-Link module with a TIPCAM1 S 3D LAP 10 mm rigid video endoscope with a 30° optic in 2D mode on a 32″ TM 330 monitor (all devices by Karl Storz (KARL STORZ SE & Co., KG, Tuttlingen, Germany). Light was provided by a cold light source POWER LED 300 (KARL STORZ SE & Co., KG, Tuttlingen, Germany). Conventional laparoscopic instruments used were two grasping forceps (dissecting and grasping forceps), scissors and a lockable needle holder (all instruments by KARL STORZ SE & Co., KG, Tuttlingen, Germany). Functional samples of motorized articulating instruments were provided by Karl Storz (KARL STORZ SE & Co., KG, Tuttlingen, Germany). They consisted of a needle holder, bipolar forceps, and bipolar scissors and were connected to a designated motor unit. All devices and instruments were CE certified.

### Questionnaire

Before starting the first exercises, participants were asked to provide general information. After completing the first E-BLUS examination, participants were handed a questionnaire asking about ergonomics of the instruments, exercise difficulties and any problems that occurred. A similar questionnaire with additional questions and participants` thoughts concerning features of the functional samples of motorized articulating instruments was administered after using ALI. All questionnaires were specifically designed for this study and provided in a digital format (SurveyGizmo, Widgix, LLC dba SurveyGizmo, 4888 Pearl East Cir., Suite 100 W, Boulder, CO 80,301) on an iPad Pro® (Apple Inc, Cupertino, California, USA) as no standardized template for this purpose existed. They were added as a supplement.

### Statistical analysis

Chi-Square test was used to evaluate group demographics. To compare exercise failure of CLI and ALI the McNemar test was applied. To assess time to completion for the E-BLUS exercises a mixed model was calculated with sequence, period, and instrument as fixed effects as well as a nested effect of participants within the randomized sequence to account for multiple measurements. Pairwise comparisons for each fixed effect were done in terms of least squares means. Paired *t*-tests were used to compare the time differences between the first and second run between the two sequences. All statistical analyses were performed using SAS® software v9.4 (SAS Institute Inc., Cary, NC, USA).

## Results

Participants in both groups were predominantly male, right-handed and between 20 and 25 years old. Most participants were either in their preclinical or clinical years. Detailed group characteristics can be found in Table 1. Statistical analysis revealed no significant differences between both groups.

### Instrument comparison (CLI vs. ALI)

Overall, using CLI was significantly faster and associated with fewer exercise failures than the new motorized ALI, defined as not completing an exercise within the given time (Tables 2 and 3). Participants failing an exercise were excluded from further statistical analysis of this exercise. When looking at the individual exercises, however, use of ALI showed no difference for the PT and CC task. Use of CLI improved speed in the NG and LS task, which reached statistical significance in NG (Table 2). Interestingly, none of the participants failed the needle guidance task with CLI, whereas 12 attempts with ALI were not successful (Table 3).

**Table 2:** Comparison of time needed to complete the E-BLUS exercises in respect to instrument used

	CLI [min]	ALI [min]	ALI-CLI (CI) [s]	*p*-value
Overall	4:27	4:50	–	0.005
Peg transfer	3:13	3:12	− 0.3 (− 17.5; 16.9)	0.97
Cutting a circle	2:44	2:40	− 4.0 (− 15.0; 7.0)	0.47
Needle guidance	5:36	7:05	88.9 (50.2; 127.6)	< 0.0001
Laparoscopic suturing	6:15	6:24	9.1 (− 35.4; 53.7)	0.68

Displayed are the least square means values as well as the difference in least square means together with 95% confidence intervals generated with the Mixed Procedure. A p-value of < 0.05 was regarded as statistically significant

*CLI* Conventional laparoscopic instruments, *ALI* articulating laparoscopic instruments

**Table 3:** Failed attempts shown for each instrument and exercise

	Failure CLI *n* = 50	Failure ALI *n* = 50	*p*-value
Peg transfer	2	2	1.00
Cutting a circle	1	1	1.00
Needle guidance	0	12	0.0005
Laparoscopic suturing	7	13	0.06

A p-value of < 0.05 was regarded as statistically significant

*CLI* Conventional laparoscopic instruments, *ALI* articulating laparoscopic instruments, *n* number of trainees

Both ALI and CLI showed the same median amount of errors in the exercises (1[1;6] vs. 1[1;9]). Also, when comparing instruments for each exercise individually median errors were comparable for PT (ALI: 1[1;5] vs. CLI: 2[1;5]), for CC (CLI: 1[1;9]) vs. ALI: 2[1;6]) and for LS (1[1;3] vs. 1[1;3]). No errors were made with either instrument in the NG task.

### Group comparison (CA vs. AC)

When comparing the mean time needed to complete all exercises of CA and AC, it did not matter which instrument was used first, as both groups showed no difference (AC: 4:49 vs. CA: 5:02 min; *p* = 0.46). Further, no statistical significance could be observed when regarding the time of exercises individually (PT: AC 3:09 vs. CA 3:15 min, *p* = 0.67; CC: AC 2:46 vs. CA 2:39 min, *p* = 0.44; LS: AC 6:22 vs. CA 6:16 min, *p* = 0.8; NG: AC 6:12 vs. CA 6:29 min, *p* = 0.56). Overall, the median amount of errors was similar between both groups (AC 1[1;5] vs. CA 1[1;9]).

As expected, a learning effect could be observed, since participants needed significantly less time to complete the exercises on the second attempt (P1: 4:55 vs. P2: 4:21 min; *p* < 0.0001). The same effect could be noticed for each of the individual exercises (PT: 3:25 vs. 2:59 min; *p* = 0.004; CC: 2:56 vs. 2:29 min; *p* < 0.0001; LS: 6:44 vs. 5:55 min; *p* = 0.03). Only the needle guidance task showed no statistical significance (6:37 vs. 6:05 min; *p* = 0.1). Interestingly, the median amount of errors was identical within both periods. (PT P1: 1[1;5] vs. P2: 1[1;4]; CC P1: 2[1;9] vs. P2: 2[1;6]; LS: P1: 1[1;3] vs. P2: 1[1;3]).

**Fig. 5:**
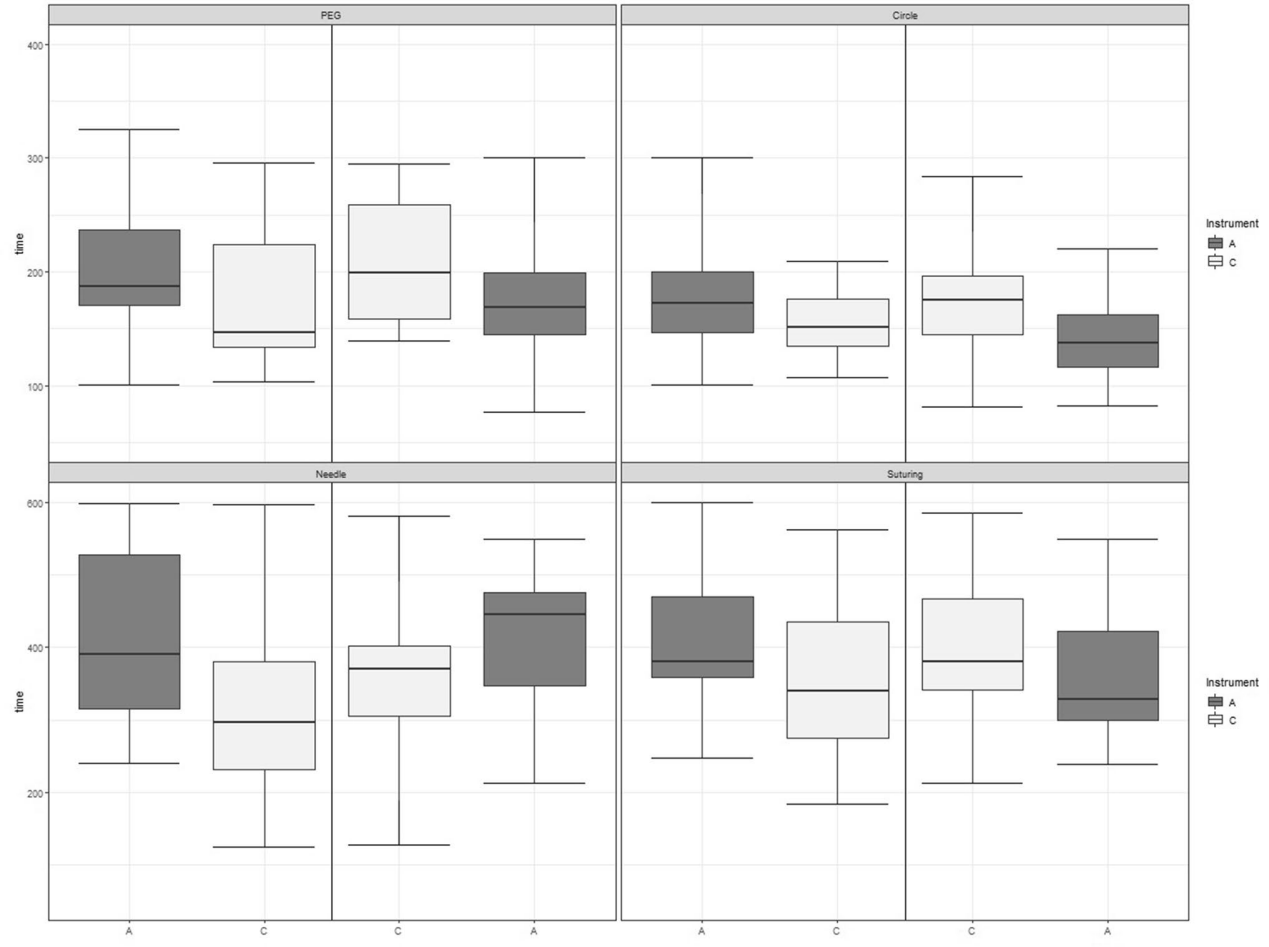
Time change from first to second attempt of E-BLUS exercises compared between groups AC and CA. Boxplots show the median time and range (in sec) of ALI and CLI for each exercise stratified by groups. Note the statistically significant slower performance for the needle guidance task at the second attempt in group CA. (A) *ALI* Articulating laparoscopic instruments. (C) *CLI *Conventional laparoscopic instruments. *PEG* Peg transfer, *Circle* Cutting a circle, *Needle* Needle guidance, *Suturing* Laparoscopic suturing

When comparing both groups regarding speed improvement from the first to the second attempt, improvement in the PT task was similar (CA: 27,5 vs AC: 26,2 s; *p* = 0.94). Although, it was not statistically significant, group CA could reduce their CC task time by an additional 6 s in the second run with ALI compared to AC (CA: 30,7 s vs. AC: 24,3 s; *p* = 0.56). Participants were clearly faster when using CLI last (group AC) in LS and NG and showed a great speed improvement from the first to the second attempt compared to CA (LS: AC 86,6 s vs. CA 51,8 s, *p* = 0.45 and NG: AC 136,2 s vs. CA − 70,1 s, *p* < 0.0001,). Interestingly, group AC was 136,2 s faster in the second run of the NG task, while no improvement for this exercise could be seen in the CA group at all. Participants using the new ALI after CLI even showed a statistically significant slower performance in the second attempt (Fig. 5).

### Usability

**Fig. 6:**
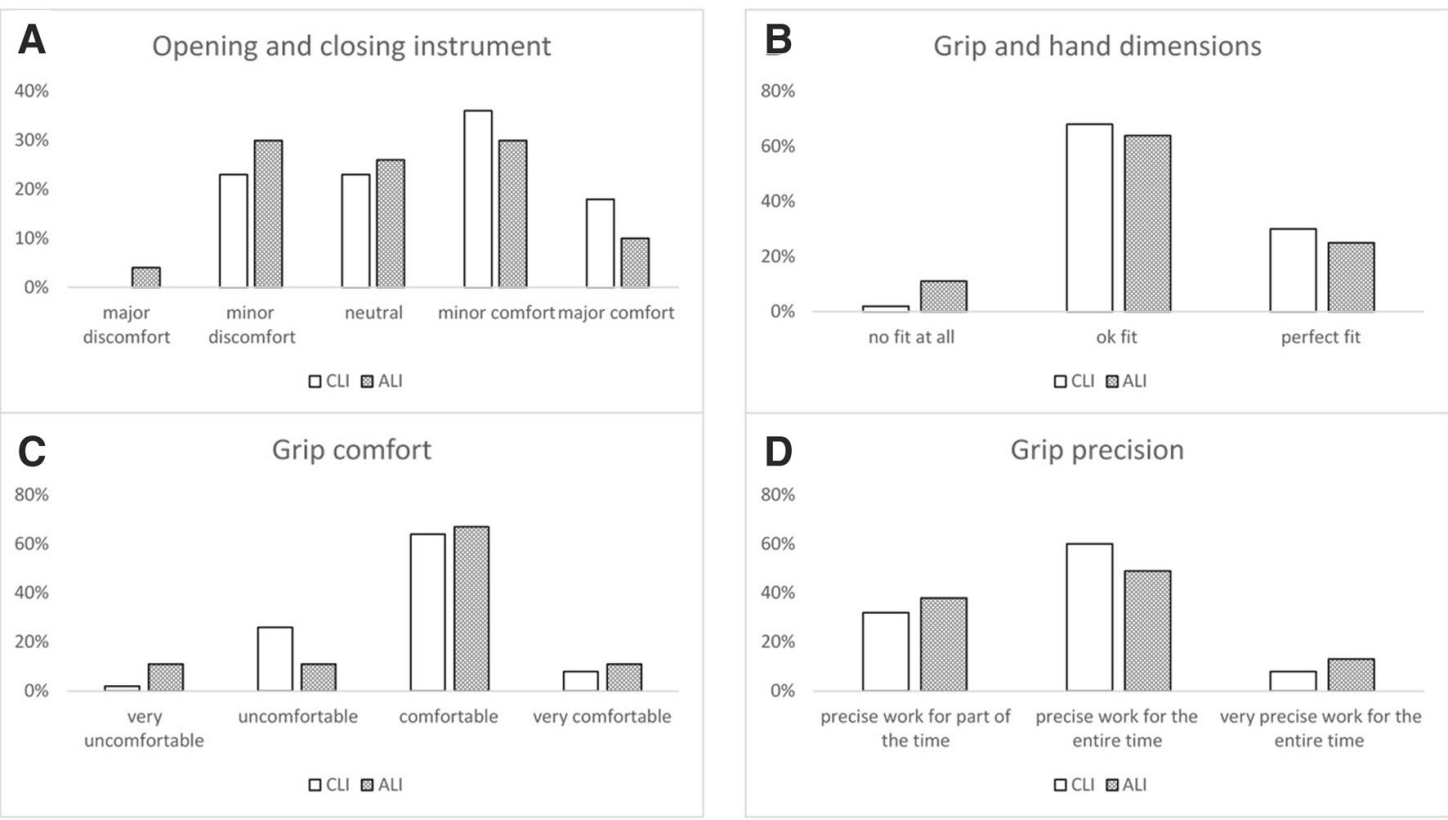
Questionnaire results of usability of instruments. Displayed are the percentages of participants’ opinions regarding opening and closing, grip and hand dimensions, grip comfort and grip precision of instruments. *CLI* Conventional laparoscopic instruments, *ALI* Articulating laparoscopic instruments

In general, participants preferred CLI over the new motorized ALI due to its lighter weight and easier handling. Opening and closing of ALI was associated with slightly more discomfort with a minority of trainees even stating major discomfort (Fig. 6A). Fit of grip and hand dimensions as well as grip comfort for the needle holder and the grasping forceps were almost similar for both instruments with a minority of students again reporting no fit at all or the grip being very uncomfortable for ALI, especially for the needle holder (Fig. 6B, C). Interestingly, grip precision was excellent in both groups with ALI allowing slightly more participants very precise work for the entire time (Fig. 6D).

61% of participants believed that additional degrees of freedom of ALI allowed easier knot tying and suturing compared to CLI, since angulating the tip from the shaft facilitated knot tying and handling of the suture. 29% disagreed, stating that ALI was too heavy and that additional degrees of freedom made the exercise more complicated. When asked if the possibility to angulate the tip of the scissors facilitated the cutting a circle task, 88% agreed stating that the biggest advantage of ALI over CLI could be seen within this exercise. 94% of trainees thought using ALI could improve working in small or narrow spaces, although some believed further experience with ALI would be needed. Further, 90% believed ALI could facilitate working around objects such as vessels. 73% agreed that ALI could offer additional benefit for knot tying and suturing in sloped planes such as the psoas muscle while 14% opposed.

Often encountered problems using ALI were heavy instruments making precise movements no longer possible, unergonomic and difficult to use ALI needle holder and the thread getting stuck in the hinge during knot tying. Overall, participants did not approve of the new ALI.

## Discussion

Over the last decades mechanical and motorized ALI have been developed to help the surgeon overcome the known obstacles of laparoscopy and, thus, making more difficult procedures possible in a minimally invasive manner. But since their introduction few studies have compared motorized ALI to CLI and their benefit is still unclear [7, 9–11, 14–16]. Some studies only compared tasks performed with motorized needle holders, like Robot DEX™ (Déxtérité Surgical, Annecy, France) or Jaimy® (Endocontrol Company, Grenoble, France) [11, 15, 16], whereas others also used several instruments of the Kymerax™ system (Terumo, Tokyo, Japan) comparable to our study [9, 10, 14, 17]. In this trial, we evaluated the performance of new motorized ALI of Karl Storz (KARL STORZ SE & Co., KG, Tuttlingen, Germany), consisting of bipolar scissors, bipolar forceps, and needle holder, in comparison to CLI. All of the above mentioned Kymerax™ studies investigated ALI’s performance in different levels of proficiency (novice, intermediate, expert) and participants per group reached up to a maximum of 25 [9, 10, 14, 17]. In contrast, we wanted to assess the new motorized ALI in a larger cohort of laparoscopic novices to create a homogenous and objective study population and, thus, no potential bias towards CLI as shown in other studies [9, 15].

Our results show that using CLI in the E-BLUS examination was significantly faster and associated with fewer exercise failures than the new motorized ALI. One explanation could be that participants felt overwhelmed by ALI’s additional degrees of freedom and instrument handling combined with the well-known intricacies of laparoscopic surgery. This is further supported by the fact that ALI showed a worse performance in more challenging tasks like NG or LS, as they require a greater depth perception and more complex instrument maneuvers.

These findings are in concordance with above mentioned research of Sieber et al. who found that participants were slower and needed more training time when using Kymerax™ compared to CLI. Their tasks were also performed in a box trainer and comparable to the ones in our study. In contrast to our results, an improved accuracy with ALI use for all exercises was recorded [10]. Our results showed a similar error rate for ALI and CLI in all tasks. It must be noted however, that Sieber et al. used a “fraying factor” for assessing instrument accuracy in cutting tasks, whereas fraying in the cutting task in our trial was not assessed as strictly leading to limited transferability of results [10].

Regarding the skills assessment in our study, it must be mentioned that only time, errors, and exercise failure were observed as foreseen in the original E-BLUS examination. However, this may have confounded our study as we were probably not able to observe the full potential of the new ALI. Especially, when evaluating more complex instruments such as motorized ALI, it would make sense to integrate tissue damage parameters such as instrument movement and interaction force into the skill assessment [18]. In a recent study about force-based laparoscopic skills training, the authors state that it is advisable to first train surgeons on efficient tissue manipulation and instrument handling before allowing them to focus on efficiency of time [19]. In this way, when training with ALI, safe tissue handling, and instrument accuracy could be evaluated more precisely.

Although there are a lot of laparoscopic tasks, we chose to use the E-BLUS examination as a validated basic laparoscopic skills curriculum in our study for reproducibility and transferability of tasks. Additionally, the tasks were selected based on the possibility to complete them with both types of instruments (ALI and CLI). Further, our study cohort only consisted of laparoscopic novices. Since E-BLUS tasks are designed to teach basic laparoscopic skills to young urology residents worldwide, they could therefore easily be transferred to our participants [13]. Furthermore, the E-BLUS examination had already been used with other ALI and showed promising results in beginners [14]. However, using only four basic laparoscopic tasks could be a limitation of our study since the E-BLUS examination was especially designed for the ergonomics of CLI and not for ALI [13]. Most advantages of motorized ALI are seen in more challenging tasks like suturing in difficult angles as seen in performing urethrovesical anastomosis or suturing on organic tissue, i.e. during bowel surgery or partial nephrectomies. Therefore, it would be interesting to explore whether the functionality of the new motorized ALI could be more accurately assessed by more demanding exercises like a “3D pick-and-place task” or ex vivo animal models in further studies [8, 11, 17, 20]. We chose laparoscopic beginners for our study cohort to ensure that we would obtain a large and consistent study population. However, some studies suggest that most advantages of ALI are only seen in experienced laparoscopist [10, 14, 21]. Therefore, it would also be of great interest to see if more experienced surgeons could expand their surgical skills by using the new ALI of our study.

Instrument usability could have played another role in differences of exercise performance in our trial. Participants generally preferred CLI over motorized ALI due to their lighter weight and easier handling. However, the ALI scissors showed promise for circular cutting tasks, as a speed improvement could be noticed in all groups and 88% of participants agreed that the biggest advantage of ALI over CLI could be seen within this exercise. A promising clinical application of ALI could therefore lie within circular cutting tasks, like partial nephrectomies, as already shown by laparoendoscopic single-site surgery using Kymerax™ instruments in a porcine model [17].

In contrast, many participants criticized the ALI needle holder for its opening/closing mechanism and uncomfortable grip. Since the ALI needle holder was used in the NG and LS task, the worse performance of ALI in those exercises could be explained. Although, participants performed LS clearly slower with ALI in our study, surprisingly 61% still believed that the additional degrees of freedom allowed easier knot tying and suturing compared to CLI. Therefore, a potential future application of ALI would be for training purposes of laparoscopic novices if instrument features could be improved further. This is supported by Zapardiel et al. who tested Kymerax™ instruments in physicians with different laparoscopic experience. He found that using ALI could help beginners in training because they needed less time for needle loading and placing a stitch with ALI compared to CLI. However, this advantage could not be seen in physicians with laparoscopic experience as they are already used to CLI [9]. In concordance, another study found that novices outscored experts in terms of net improvement in performance with articulating instruments. To speed the learning process, the authors suggest the use of articulating instruments at an early stage of surgical training [21]. Thus, follows that ALI seem to have their own learning curve and require skills distinct form conventional laparoscopy or open surgery. However, one should keep in mind that ALI are not designed to be a replacement of CLI, but should rather be seen as an add-on to refine laparoscopic capabilities [7]. Therefore, laparoscopic training with CLI and ALI should ideally be combined in a new multi-modality training curriculum to ensure an optimal training benefit with both types of instruments. One approach could be comparable to the multi-modality training for laparoscopic cholecystectomy which has already been explored by Kowaleski and colleagues [22].

## Conclusion

In conclusion, using new motorized ALI in a basic laparoscopic skills curriculum by beginners was associated with a slower performance and more exercise failures compared to CLI. One explanation could be that laparoscopic novices felt overwhelmed by the additional features of motorized ALI and ALI have a distinct learning curve that needs to be addressed. However, participants believed that additional degrees of freedom allowed easier knot-tying, suturing, and cutting compared to CLI. Therefore, ALI should be used for laparoscopic training purposes, ideally combined with conventional laparoscopic training in a new curriculum. Enhancing ergonomics and reducing weight could help to further improve ALI’s user-friendliness.

## Electronic supplementary material

Below is the link to the electronic supplementary material.

Supplementary file1 (DOCX 27 kb)

## Abbreviations

ALI: Articulating laparoscopic instrument(s)
CC: E-BLUS exercise “cutting a circle”
CLI: Conventional laparoscopic instrument(s)
E-BLUS: European training in basic laparoscopic urological skills
LS: E-BLUS exercise “laparoscopic suturing”
NG: E-BLUS exercise “needle guidance”
PT: E-BLUS exercise “peg transfer
