# Supplementary material for: Evaluation of new motorized articulating laparoscopic instruments by laparoscopic novices using a standardized laparoscopic skills curriculum
Source: Surg Endosc. 2020 Oct 20;35(2):979–88. doi: 10.1007/s00464-020-08086-2 (PMC7819923; doi:10.1007/s00464-020-08086-2)
Supplement: Supplementary file 1 — Supplementary file1 (DOCX 27 kb) [file 464_2020_8086_MOESM1_ESM.docx]

**Supplementary Material**

**regarding the manuscript “Evaluation of new motorized articulating laparoscopic instruments by laparoscopic novices using a standardized laparoscopic skills curriculum”**

**Detailed explanation of the performed exercises:**

*Warm-up exercises*

To familiarize participants with the laparoscopic working station and instruments three warm-up exercises were conducted before starting the E-BLUS examination (Fig. 3). The exercise was demonstrated by the observer (D.U.) and any questions regarding instrument handling and exercise specifications were answered.

During the first warm-up exercise participants had to stretch a rubber band between two hooks after threading the band through two rings. Participants had to start at the left hook and repeat the procedure from the opposite site after the first rubber band stretch (Fig. 3, A). The second warm-up exercise asked for five horizontally arranged matches with tips pointing outward to be transferred into a match box. Afterwards, the matches were transferred to the left side of the board in the same manner. They were then placed back into the box before being returned to the starting position (Fig. 3, B). For the third warm-up exercise participants had to tie a surgeon’s knot. The knot tying technique can be found in the description of the laparoscopic suturing task (Fig. 3, C).

*E-BLUS examination*

Task 1: Peg transfer (PT)

Six pegs were mounted in prefixed positions (Fig. 2, A). First, pegs had to be transferred from the left to the right by passing them in the air to the other instrument. Pegs were then returned to their original position the same way. The exercise was started with the non-dominant hand. Therefore, directions changed for left-handed participants from right to left. For CLI a broad clamp was used in the non-dominant hand and an overholt clamp was used in the dominant hand. For ALI the overholt clamp was replaced with the articulating bipolar forceps. Dropping a peg in the actual examination would lead to the exercise being suspended and the task to be failed. We chose to count this as an error and asked participants to return the peg to the position from which it had been picked up prior to being dropped.

Task 2: Cutting a circle (CC)

Setup consisted of a double layer gauze upon which two concentric circles had been drawn (Fig. 2, B). Participants were asked to cut out the inner circle while staying within the space between the two lines. The position of the first cut and the direction in which the circle was cut out were left to the participants discretion. For CLI scissors and an overholt clamp were used. For ALI bipolar scissors and an overholt clamp were used. Scissors had to be used with the dominant hand. Cutting into one of the lines or outside was defined as a mistake.

Task 3: Needle guidance (NG)

The aim of this task was to thread a suture through several small rings on a prefixed “snakelike” pattern (Fig. 2, C). For this exercise participants were given two needle holders for CLI, with one being replaced by an articulating needle holder for ALI.

If a ring was left out or entered from the wrong side, this was counted as a mistake. When the needle sprung out of the visual field, the time was stopped and restarted once the needle had been repositioned correctly within the needle holder.

Task 4: Laparoscopic suturing (LS)

In the final task a surgical suture had to be performed (Fig. 2, D). A pre-cut penrose drain with two black dots and a longitudinal cut (3B Scientific GmBH, Hamburg, Germany) was secured to an exercise board. A curved needle mounted with a 2-0 Vicryl suture (Covidien plc, Dublin, Ireland), pre-cut to 20 cm, was positioned on the right side of the drain. The suture needed to be stitched through the black dots and then knotted tightly by one double and two single throws. Finally, the suture was cut, leaving a secure surgical knot.

For this exercise two CLI needle holders were used, with one replaced by an articulating needle holder for ALI. Stitching beyond one millimeter of one or both black dots, leaving a gap in the slit of the drain after tying the knot, a slipping knot and the penrose drain coming loose from the board or being torn apart were counted as mistakes.

**Questionnaire (Part 1-4)**

#### **Part 1: Demographic information of participants (administered before starting the experiment)**

#### 1) Gender

( ) Male

( ) Female

### 2) Age

_________________________________________________

#### 3) Semester

( ) 1st Semester

( ) 2nd Semester

( ) 3rd Semester

( ) 4th Semester

( ) 5th Semester

( ) 6th Semester

( ) 7th Semester

( ) 8th Semester

( ) 9th Semester

( ) 10th Semester

( ) “PJ” (practical year = last year of training)

( ) other (resident, attending physician)

#### 4) Are you left-handed or right-handed?

( ) left-handed

( ) right-handed

#### 5) How would you characterize your interests and which type matches you best?

[ ] interested in mathematics

[ ] interested in science

[ ] interested in languages and/or humanities

[ ] interested in crafts

[ ] interested in arts/creative

### 6) Do you have any prior experience in laparoscopy?

_________________________________________________

#### 7) Does any of the following hobbies match you?

( ) electronic gaming consoles (Playstation, Xbox, Wii, PC,...)

( ) musical instrument

( ) knitting, pottery

( ) suture courses

( ) modelling (constructing of models)

### 8) Do you currently have an occupation that demands increased levels of manual activities (lots of structural precision work, typing, etc.) If **yes**, please name your current occupation.

_________________________________________________

**Part 2: Instrument features and usability (administered after the first run with either ALI or CLI)**

#### 9) How much comfort/discomfort do you experience when opening and closing the instruments?

[ ] absolute discomfort

[ ] little discomfort

[ ] neutral

[ ] slight comfort

[ ] pronounced comfort

### 10) How much comfort/discomfort does the rotation function of the instrument tip give you? (please fill in this question only for articulating instruments)

____________________________________________

#### 11) To what extent do the dimensions of the handles correspond to your hands?

( ) Do not match my hands at all

( ) Roughly match my hands

( ) Matches my hands exactly

#### 12) How comfortable is the handle in your hand?

( ) very comfortable

( ) uncomfortable, disturbing feeling when holding, but does not make work more difficult

( ) comfortable feeling when holding

( ) very comfortable

#### 13) How precisely can you work with the handle?

( ) a precise work with the handle is not possible

( ) precise working is partly possible with the handle

( ) a precise work is possible with the handle over the whole time

( ) a very precise work is possible with the handle over the whole time

### 14) What is your general impression of the handle? (Please specify between pistol and needle holder handle)

____________________________________________

#### 15) Have you experienced any of the following problems during or after the laparoscopic exercises? (please mark the correct answer with an x)

| ­ | No | Light | Heavy |
| --- | --- | --- | --- |
| Neck pain |  |  |  |
| Neck stiffness |  |  |  |
| Shoulder/arm pain |  |  |  |
| Shoulder/arm stiffness |  |  |  |
| Wrist stiffness |  |  |  |
| Back pain |  |  |  |
| Back rigidity |  |  |  |
| Headaches |  |  |  |
| Tiredness, irritability, exhaustion |  |  |  |
| Excessive tachycardia/sweating/tremor |  |  |  |
| Unarticulated/difficult manipulation of the instruments |  |  |  |
| Inability to perform fine or precise exercises |  |  |  |

#### 16) Please rank the exercises according to their perceived difficulty by giving them stars from 1-7.

|  | Level of difficulty (1=simplest, 7=most difficult) |
| --- | --- |
| Warm-up exercise 1  (rubber band transfer) | _________________________________________________ |
| Warm-up exercise 2  (match transfer: 90° rotation) | _________________________________________________ |
| Warm-up exercise 3 (Knot tying) | _________________________________________________ |
| Peg Transfer | _________________________________________________ |
| Cutting a circle | _________________________________________________ |
| Needle guidance | _________________________________________________ |
| Laparoscopic suturing | _________________________________________________ |

### 17) Do you think that the height of the workstation is comfortable for laparoscopy? If not, should it be possible to raise or lower the station from its current position?

____________________________________________

### 18) Do you think that ergonomic conditions in the operating theater play a role?

____________________________________________

19) Did you have the feeling that trocar positioning gives you adequate degrees of freedom and leeway in handling the instruments?

____________________________________________

**Part 3: Instrument features and usability (administered after the second run with either ALI or CLI)**

20) How much comfort/discomfort do you feel when opening and closing the instruments?

[ ] absolute discomfort

[ ] little discomfort

[ ] neutral

[ ] light comfort

[ ] pronounced comfort

21) How much comfort/discomfort does the rotation function of the instrument tip give you? (please fill in this question only for articulating instruments)

____________________________________________

22) To what extent do the dimensions of the handles correspond to your hands

( ) Do not match my hands at all

( ) Roughly match my hands

( ) Matches my hands exactly

23) How comfortable is the handle in your hand?

( ) very comfortable

( ) uncomfortable, disturbing feeling when holding, but does not make work more difficult

( ) comfortable feeling when holding

( ) very comfortable

24) How precisely can you work with the handle?

( ) a precise work with the handle is not possible

( ) precise working is partly possible with the handle

( ) a precise work is possible with the handle over the whole time

( ) a very precise work is possible with the handle over the whole time

25) What is your general impression of the handle? (Please specify between pistol and needle holder handle)

____________________________________________

26) Have you experienced any of the following problems during or after the laparoscopic exercises? (please mark the correct answer with an x )

| ­ | No | Light | Heavy |
| --- | --- | --- | --- |
| Neck pain |  |  |  |
| Neck stiffness |  |  |  |
| Shoulder/arm pain |  |  |  |
| Shoulder/arm stiffness |  |  |  |
| Wrist stiffness |  |  |  |
| Back pain |  |  |  |
| Back rigidity |  |  |  |
| Headaches |  |  |  |
| Tiredness, irritability, exhaustion |  |  |  |
| Excessive tachycardia/sweating/tremor |  |  |  |
| Unarticulated/difficult manipulation of the instruments |  |  |  |
| Inability to perform fine or precise exercises |  |  |  |

27) Please rank the exercises according to their perceived difficulty by giving them stars from 1-7.

|  | Level of difficulty (1=simplest, 7=most difficult) |
| --- | --- |
| Warm-up exercise 1  (rubber band transfer) | _________________________________________________ |
| Warm-up exercise 2  (match transfer: 90° rotation) | _________________________________________________ |
| Warm-up exercise 3 (Knot tying) | _________________________________________________ |
| Peg Transfer | _________________________________________________ |
| Cutting a circle | _________________________________________________ |
| Needle guidance | _________________________________________________ |
| Laparoscopic suturing | _________________________________________________ |

28) Do you think that the height of the workstation is comfortable for laparoscopy? If not, should it be possible to raise or lower the station from its current position?

____________________________________________

29) Do you think that ergonomic conditions in the operating theater play a role?

____________________________________________

30) Did you have the feeling that trocar positioning gives you adequate degrees of freedom and leeway in handling the instruments?

____________________________________________

**Part 4: Application of ALI and questions regarding laparoscopic training (administered after completion of the experiment)**

31) Do you think that the additional degrees of freedom of the articulating instruments offer you an easier knot tying and suturing compared to conventional instruments?

____________________________________________

32) Do you think that the possibility of angling the scissors makes it easier to cut the circle?

____________________________________________

33) Do you think that the use of articulating instruments makes it easier to work in confined spaces? (e.g. in small corners)

____________________________________________

34) Do you think that the use of articulating instruments would facilitate working around obstacles (vessels, organs, ...)?

____________________________________________

35) Do you think that articulating instruments are superior when knot tying and suturing in the bevel? (Example: you need to tie a knot on a sloping surface, such as the psoas muscle) ____________________________________________

36) Do you think articulating instruments could be a less expensive alternative to the DaVinci robot?

____________________________________________

37) Have you experienced increased problems with the articulating instruments? If so, in which exercise and how did these problems manifest themselves?

____________________________________________

____________________________________________

38) Do you think there should be simulation-based training of laparoscopic skills as part of the surgical/urological specialist training?

____________________________________________

39) If such training existed, would you participate in it?

( ) Yes

( ) No

40) If yes, how much time would you be willing to spend on this? (in minutes to hours/per day/per week)

_________________________________________________

41) Do you think there should be a free period during residency for such a training?

( ) Yes

( ) No

42) Do you think that such training would give you more confidence during the actual operation?

( ) Yes

( ) No

43) Do you think there should be basic courses for learning laparoscopic skills during medical school to give students an earlier insight into the practical side of surgery?

( ) Yes

( ) No

44) Do you think that you would also take advantage of simulation-based exercise offers outside your working hours?

( ) Yes

( ) No

45) Do you think that simulation-based training should be used as a parameter for quality assurance and the learning level of operators?

( ) Yes, I am of the opinion

( ) No, I do not think so

46) If you have ticked No, please give reasons for your statement.

____________________________________________

47) Do you think a surgeon could benefit from simulation-based warm-up exercises before surgery?

( ) Yes

( ) No

48) Please justify your answer from the previous question.

____________________________________________

49) Do you believe simulation-based training represents the future of surgical training?

____________________________________________

50) Are you interested in further studies of this kind with other such topics (laparoscopic instruments, laparoscopic surgery, etc.)?

_________________________________________________

51) If yes, would you please write your email address here so we can contact you?

_________________________________________________

Thank You!
